# Antimicrobial Usage and Resistance in Makkah Region Hospitals: A Regional Point Prevalence Survey of Public Hospitals

**DOI:** 10.3390/ijerph19010254

**Published:** 2021-12-27

**Authors:** Abdul Haseeb, Hani Saleh Faidah, Manal Algethamy, Saleh Alghamdi, Ghaidaa Ali Alhazmi, Afnan Owedah Alshomrani, Bashair Rjyan Alqethami, Hind Saeed Alotibi, Maali Zayed Almutiri, Khawlah Saad Almuqati, Amjad Abdullah Albishi, Mahmoud Essam Elrggal, Ahmad Jamal Mahrous, Asim Abdulaziz Khogeer, Zikria Saleem, Muhammad Shahid Iqbal, Aziz Sheikh

**Affiliations:** 1 Department of Clinical Pharmacy, College of Pharmacy, Umm Al Qura University, Makkah 21955, Saudi Arabia; merggal@uqu.edu.sa (M.E.E.); ajmahrous@uqu.edu.sa (A.J.M.); 2 Department of Microbiology, Faculty of Medicine, Umm Al Qura University, Makkah 21955, Saudi Arabia; hsfaidah@uqu.edu.sa; 3 Department of Infection Prevention and Control Program, Alnoor Specialist Hospital Makkah, Makkah 24382, Saudi Arabia; mmalgethamy@moh.gov.sa; 4 Department of Clinical Pharmacy, Faculty of Clinical Pharmacy, Al Baha University, Al Baha 65779, Saudi Arabia; saleh.alghamdi@bu.edu.sa; 5 Departments of Pharmacy, King Abdullah Medical City, Ministry of Health, Makkah 21955, Saudi Arabia; Ph.ghaidaa@outlook.sa; 6 King Abdul Aziz Medical City, WR, Jeddah, Ministry of National Guard, Jeddah 21423, Saudi Arabia; Shomraniaf@gmail.com; 7 Maternity and Children Hospital, Makkah 21955, Saudi Arabia; balqethami@moh.gov.sa; 8 Prince Meshari Bin Saud- General Baljarshi Hospital, Al Baha 65779, Saudi Arabia; Ph.hind22saeed@gmail.com; 9 Sulaiman AlHabib Medical Group, Jeddah 22230, Saudi Arabia; Ph.maalizayed@gmail.com; 10 King Faisal Specialist Hospital and Research Centre (Gen. Org.), Riyadh 11211, Saudi Arabia; khawlah.almuqati@gmail.com; 11 Maternity and Children Hospital, Bishah 24213, Saudi Arabia; amabalbishi@moh.gov.sa; 12 Plan and Research Department, General Directorate of Health Affairs of Makkah Region, Ministry of Health, Makkah 21955, Saudi Arabia; akhogeer@moh.gov.sa; 13 Medical Genetics Unit, Maternity and Children Hospital, Makkah Healthcare Cluster, Ministry of Health, Makkah 21955, Saudi Arabia; 14 Department of Pharmacy Practice, Faculty of Pharmacy, New Campus, The University of Lahore, Lahore 54000, Pakistan; xikria@gmail.com; 15 Department of Clinical Pharmacy, College of Pharmacy, Prince Sattam Bin Abdulaziz University, Alkharj 11942, Saudi Arabia; M.javed@psau.edu.sa; 16 Usher Institute, Old Medical School, The University of Edinburgh, Teviot Place, Edinburgh EH8 9AG, UK; Aziz.Sheikh@ed.ac.uk

**Keywords:** antimicrobial consumption, antimicrobial resistance, point prevalence survey, hospital, Saudi Arabia

## Abstract

(1) Background: Inappropriate use of antimicrobials and subsequently rise of antimicrobial resistance (AMR) remains a major public health priority. Over-prescribing of broad-spectrum antibiotics is one of the main contributing factors for the emergence of AMR. We sought to describe antimicrobial prescribing trends among patients in public hospitals in Makkah hospitals. (2) Method: We undertook a point prevalence survey (PPS) in six hospitals in Makkah, Saudi Arabia, from January 2019 to July 2019. The survey included all the inpatients receiving antimicrobials on the day of PPS. Data was collected using the Global point prevalence survey (PPS) tool developed by the University of Antwerp, Belgium. (3) Results: Of 710 hospitalized patients, 447 patients (61.9%) were treated with one or more antimicrobials during the study period. The average bed occupancy among six hospitals was 74.4%. The majority of patients received antimicrobials parenterally (90.3%). Of the total prescribed antimicrobials, 415 (53.7%) antimicrobials were used in medical departments, 183 (23.7%) in surgical departments, and 175 (22.6%) in ICUs. Pneumonia (17.3%), skin and soft tissue infections (10.9%), and sepsis (6.6.%) were three common clinical indications. Ceftriaxones were the most commonly used antibiotics that were prescribed in 116 (15%) of patients, followed by piperacillin, with an enzyme inhibitor in 84 (10.9%). (4) Conclusion: There was a high prevalence of antibiotic use in the hospitals of Makkah, which could be a potential risk factor for the incidence of resistant strains, particularly MRSA infection. Public health decision-makers should take these findings into consideration to update national policies for antibiotic use in order to reduce the risks of further increases of AMR.

## 1. Introduction

Antimicrobials are commonly used to treat or prevent bacterial infections. Some antimicrobials may be used inappropriately—for example, if not indicated or suboptimal selection, dose, route of administration, or duration [1,2]. Antimicrobial selection pressure contributes to the emergence and spread of antimicrobial resistance (AMR) [3,4]. In the last few decades, the inappropriate use of antimicrobials and the rise of AMR has emerged as major global public health concern [5]. In an attempt to address the risks posed by AMR, the World Health Organization (WHO) recommends the initiation of antimicrobial stewardship programs (ASPs) to identify and monitor antimicrobial use and reduce the burden of AMR [6,7]. Economically developed countries have started implementing ASP to resolve this issue; however, in economically developing countries, the scenario is quite different. In the Gulf region, there is still a lack of knowledge and worrying attitudes and belief concerning antimicrobial use [8].

Saudi Arabia is the cornerstone of pilgrimage for Muslims from all over the world for Hajj/Umrah, with these religious rites centered on the holy city of Makkah. The extreme congestion of Saudi and non-Saudi populations results in the occurrence and spread of various infectious diseases, specifically when the healthcare system is not well-established [9,10]. A national point prevalence survey (PPS) carried out in 26 hospitals in Saudi Arabia reported that 45.7% of antimicrobials are used in surgical departments, and 59.6% of patients received at least one antimicrobial in Makkah hospital, representing the fourth highest antimicrobial usage nationally [11]. Another PPS was carried out in one hospital in Jeddah, Saudi Arabia reporting high antimicrobial usage in this region [12,13]. The uncontrolled usage of antimicrobials has the potential to contribute to the spread of antimicrobial-resistant agents nationally, as well as globally [14]. Information on antimicrobial use and AMR from PPSs could be used to design, implement and assess the effects of antimicrobial policies [15]. Limited data regarding antimicrobial use by using PPS tool is available in hospitals of the Makkah region dealing with pilgrims specifically. Moreover, data highlighting antimicrobial use and AMR among Makkah residents are not available. Therefore, this study was carried out to identify antimicrobial prescribing practices among patients in public and private hospitals in Makkah hospitals.

## 2. Materials and Methods

### 2.1. Study Design and Settings

A multicenter PPS of antimicrobial use was carried out among inpatients at six hospitals of Makkah, Saudi Arabia. The global point prevalence survey method was adopted, which aims to provide a standardized method for surveillance of antimicrobial use and to assess the quality of antimicrobial prescribing [12,13,16,17]. This survey was undertaken in six hospitals in the Makkah region.

### 2.2. Sampling and Recruitment of Hospitals

Participation of hospitals was voluntary. Different public sector hospitals were invited to participate in this survey. In case of refusal of the first hospital, the next health care setting was selected. The health care facilities providing acute care facilities were included. Whereas the health care facilities providing only nursing care, rehabilitation centers, or psychiatric centers were not included.

### 2.3. Inclusion and Exclusion Criteria

All the inpatients present in the wards before 8:00 am and not discharged on the day of the survey were included in this study. Data was collected from those who received at least one antimicrobial for at least one clinical condition or prophylaxis at the time of the survey. Outpatients and patients undergoing any surgery at the time of the survey were excluded. Patients who did not receive antimicrobials or were admitted to long-term care wards, emergency departments, and day-care wards (e.g., renal dialysis ward) were also excluded from this survey.

### 2.4. Case Definition and Data Collection

Antibiotics were classified according to the anatomical therapeutic chemical (ATC) classification system developed by the WHO Collaborating Centre for Drug Statistics Methodology in Oslo, Norway. Only antimicrobials for systemic use (ATC J01) were included in the survey. Two forms were used to collect data: one to collect about the relevant ward/department and the second to gather information related to patients who were on systematic antimicrobial agents during PPS. The data collected from ward/department included: the type of ward/department (medical, surgical, or intensive care unit), the total number of beds, all admitted patients, and the number of patients on antimicrobials. The patient form was designed to obtain information about patients and the antibiotics they were taking. The data obtained included: the patient’s characteristics; details of their prescribed antimicrobials, such as drug name, unit dose, frequency, and reasons for prescribing. Compliance with clinical guidelines was also assessed. Compliance was measured by assessing prescribing patterns against institutional antimicrobial prescribing guidelines. All data collectors had a training session about key objectives and data collection approach for this survey. A pilot survey was carried out by collecting data from 5 patients to review the quality of protocol. Data were collected from January 2019 to July 2019.

### 2.5. Timeline

Included hospitals had to be completed PPS within two to three weeks from the first day of data collection. To minimize the effect of movement of patients between wards and within hospitals, each ward was completely surveyed within one day. Due to limited staff availability, no survey was carried out during weekends or bank holidays.

## 3. Results

A total of six hospitals with 950 beds were surveyed. Of 710 hospitalized patients, 447 patients (61.9%) were treated with one or more antimicrobials during the study period. The demographics and clinical characteristics of inpatients are described in Table 1. The average bed occupancy among six hospitals was 74.4%. The total number of prescribed antimicrobials was 774, and of these, 90.3% of antimicrobials were administered parenterally, and 9.7% of antimicrobials were given orally. The majority of patients (52.0%) received antimicrobials for community-acquired infections. Most of the surveyed patients were admitted to medical departments (53.7%), followed by surgical wards (23.7%) and intensive care units (ICUs) (22.6%). Additionally, empirical treatment (85.4%) was more common. 

Compliance with clinical guidelines was also evaluated; most of the hospitals were compliant to guidelines, while the highest compliance rate was in H6 hospitals, with a 78.9% compliance rate, while the least compliance rate was shown in H2 hospitals. The use of the main antimicrobial classes was also evaluated and described in Table 2. The total number of antibacterial agents prescribed for systemic use (J01) was 744, which represents 96.2% of the total antimicrobial agents used. The antimicrobials included J01A;1.8%, J01B;0.1%, J01C;19.5%, J01D; 36.9%, J01E; 0.4%, J01F;10%, J01G; 3%, J01M;9.6%, J01X;15%.

**Figure 1:**
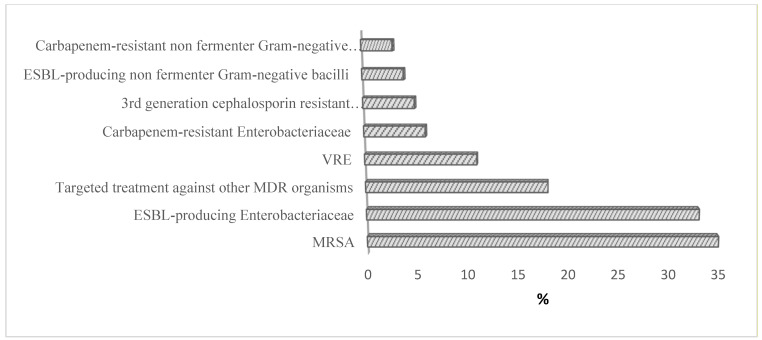
Common pathogens reported in hospitals.

The most common indications and used antimicrobials are reported in Table 3. The most common indication for antibiotics prescription was pneumonia, with 134 prescriptions (17.3%), while the least prevalent indication was both the cardiovascular system and prophylactic gastrointestinal tract infections, representing 25 prescriptions (3.2%). Ceftriaxone was the most commonly used antibiotic, which was prescribed in 116 (15%) of patients, followed by piperacillin, with an enzyme inhibitor in 84 (10.9%) of the prescriptions. At the same time, the least prescribed antibiotic was ciprofloxacin in 25 (3.2%) prescriptions. As for the type of bacteria isolated from cultures, Methicillin-resistant staphylococcus aureus (MRSA) was the most frequently isolated bacterial strain in 35.0% of cultures, while carbapenemase-producing gram-negative bacilli were the least common isolated bacterial strain, which was isolated in less than 5% of the cultures as shown in Figure 1.

## 4. Discussion

Antimicrobial resistance has been a global hazard over the past two decades. It has been attributed to the abuse of antibiotics in both in-hospital and outpatients’ settings, particularly broad-spectrum antibiotics. It has been revealed that almost up to 40% of hospitalized patients received antibiotics prescriptions, which are non-compliant with clinical guidelines [11]. Additionally, the excessive use of antibiotics for inappropriate indications or incomplete durations can also increase the burden of antimicrobial resistance. Hence, point prevalence studies can spot the light on areas of misuse and guide in developing national antibiotic use strategies [4]. 

The study demonstrated that up to 61.9% of patients were treated with at least one antimicrobial. This prevalence rate of antimicrobial use is higher than other PPS studies conducted in different regions of the world, including Germany (25.5%), Scotland (28.3%), Norway (16.6%), and Canada (17.3%) [18,19,20,21]. However, this is similar to some of the PPS surveys reported in LMICs, particularly the Asian countries, including Pakistan (77.6%), China (75.3%), and India (57.4%) [16,22,23]. Community-acquired infection was the main indication for antibiotics prescription, specifically pneumonia (17.3%); this is similar though to the study reported in Japan (20%) and Pakistan (34.2%), where most of the patients received antimicrobials for the treatment of community-acquired infections [16,24]. The majority of the patients received antimicrobials parenterally that were similar to previously reported studies [20].

A difference in prescribing patterns was observed among these hospitals. The number of antimicrobials per patient was 1.76, which is almost similar to the study conducted in Nigeria (1.77), Japan (1.66), and Pakistan (1.64) [23,25,26]. The predominantly prescribed antimicrobials were cephalosporins which is similar to other previous studies conducted in Eastern Asia, Southern Asia, Africa, and Northern Europe [16,20,22,23,24,25]. Another study conducted in Saudi Arabia also reported that cephalosporins were the most frequently prescribed antimicrobial class in 26 MOH hospitals [11]. The antimicrobial usage was higher in medical departments (53.7%). However, in Turkey, antimicrobial use was higher in surgical ICU wards (81%) [26]. In South Africa, 83% of the prescriptions were modified based on culture results, while in the present study, only 30% of culture reports were found from the six hospitals [27]. 

The most recent point prevalence survey was performed in Ghana to examine antibiotic use as part of an antibiotic stewardship program in two hospitals [28]. It showed compliance with guidelines between 50–67%, which was lower than that measured in the present study. However, similar to the present study, most of the prescribed antibiotics were used systemically and on an empirical basis. Improvements in prescribing practices are required by developing evidence-based guidelines, improving laboratories, and retaining prescribers on the importance of empiric or targeted therapy. The present study could be the basis for initiating and implementing the ASPs for different scales of the hospital. Nevertheless, the present study had some limitations. All the included hospitals were only in Makkah city, which makes extrapolating the findings to other cities in Saudi Arabia difficult. Secondly, data of some patients were incomplete to assess the diagnosis of infectious disease, which could affect the PPS results. This is considered the first study in Makkah city to evaluate antibiotic use through a point prevalence survey in multiple hospitals.

## 5. Conclusions

We found a very high prevalence of antibiotic use in Makkah hospitals. Public health decision-makers in Makkah city should take these findings into consideration to update national policies for antibiotic use in order to find ways of curbing the growth of AMR. These policies are particularly essential for community-acquired infections. Additionally, similar studies are required in other areas in Saudi Arabia to provide comparative national estimates for other parts of the Kingdom.

## Figures and Tables

**Table 1:** Overall antibiotic use prevalence.

Characteristics N (%)	H1	H2	H3	H4	H5	H6	Total	Range
Hospital Type								
Total beds	175	180	118	174	283	20	950	20–283
Hospitalized patients	122 (69.7)	152 (84.4)	96 (81.3)	121 (69.5)	202 (71.3)	15 (75.0)	710 (74.7)	69.5–84.4
Treated patients	79 (64.7)	82 (53.9)	67 (69.8)	57 (47.1)	141 (69.8)	14 (93.3)	440 (61.9)	47.1–93.3
Prescribed antibiotics (per patient)	140 (1.7)	150 (1.8)	129 (1.9)	106 (1.8)	229 (1.6)	19 (1.3)	773 (1.7)	1.3–1.9
Departments								
Surgical Department	32 (22.9)	25 (16.7)	33 (25.6)	12 (11.3)	81 (35.4)	-	183 (23.7)	11.3–35.4
Medical Department	74 (52.9)	125 (83.3)	39 (30.2)	71 (67.0)	106 (46.3)	-	415 (53.7)	30.2–83.3
Intensive Care Unit	34 (24.3)	-	57 (44.2)	23 (21.7)	42 (18.3)	19 (100)	175 (22.6)	0–100
Gender								
Male	74 (52.9)	42 (28.0)	70 (54.3)	70 (66.0)	143 (62.4)	10 (52.6)	409 (52.9)	28.0–66.0
Female	66 (47.1)	105 (70.0)	54 (41.9)	36 (34.0)	86 (37.6)	9 (47.4)	356 (46.1)	34.0–70.0
Route of administration								
Oral	13 (9.3)	15 (10.0)	5 (3.9)	12 (11.3)	25 (10.9)	5 (26.3)	75 (9.7)	3.9–26.3
Parenteral	127 (90.7)	135 (90.0)	124 (96.1)	94 (88.7)	204 (89.1)	14 (73.7)	698 (90.3)	73.7–96.1
Indication								
Community-acquired infection	56 (40.0)	132 (88.0)	37 (28.7)	44 (41.5)	114 (49.8)	19 (100.0)	402 (52.0)	40.0–100.0
Hospital-acquired infection	73 (52.1)	18 (12.0)	27 (20.9)	40 (37.6)	60 (26.2)	-	218 (28.1)	0.0–52.1
Medical prophylaxis	-	-	-	2 (1.9)	5 (2.2)	-	7 (0.9)	0.0–2.2
Surgical prophylaxis (single dose)	3 (2.1)	-	4 (3.1)	5 (4.7)	7 (3.1)	-	19 (2.5)	0.0–4.7
Surgical prophylaxis (one day)	4 (2.9)	-	13 (10.1)	4 (3.8)	11 (4.8)	-	32 (4.1)	0.0–10.1
Surgical prophylaxis (>1 day)	4 (2.9)	-	1 (0.8)	9 (8.5)	26 (11.4)	-	40 (5.2)	0.0–11.4
Others	-	-	47 (36.5)	2 (1.9)	6 (2.6)	-	55 (7.1)	0.0–36.5
Treatment								
Empirical therapy	108 (77.1)	143 (95.3)	107 (82.9)	89 (84.0)	194 (84.7)	19 (100.0)	660 (85.4)	77.1–100.0
Targeted therapy	32 (22.9)	7 (4.7)	22 (17.1)	17 (16.0)	35 (15.3)	-	113 (14.6)	0.0–22.9
Guideline’s compliance								
Yes	70 (50.0)	12 (8.0)	54 (41.9)	17 (16.0)	57 (24.9)	14 (78.9)	225 (29.1)	8.0–78.9
No	65 (46.4)	-	1 (0.8)	10 (9.4)	31 (13.5)	4 (21.1)	111 (14.4)	0.0–46.4
NA	-	-	25 (19.4)	4 (3.8)	25 (10.9)	-	54 (7.0)	0.0–19.4
NI	5 (3.6)	138 (92.0)	49 (38.0)	75 (70.8)	116 (50.7)	-	383 (49.5)	0.0–92.0
Stop date documented	35 (25.0)	57 (38.0)	29 (22.5)	49 (46.2)	74 (32.3)	16 (84.2)	260 (33.6)	22.5–84.2
Reason on notes	79 (56.4)	54 (36.0)	84 (65.1)	56 (52.8)	150 (65.5)	16 (84.2)	439 (56.8)	36.0–84.2
Culture Reports	32	7	22	34	37	0	132	0–37

**Table 2:** Use the prevalence of main antibiotics classes.

Antibiotics	*n* (%)
ANTIBACTERIALS FOR SYSTEMIC USE (J01)	744 (96.2)
Tetracyclines (J01A)	14 (1.8)
Amphenicols (J01B)	1 (0.1)
Penicillins (J01C)	151 (19.5)
Cephalosporins and Penams (J01D)	285 (36.9)
Sulfonamides and trimethoprim (J01E)	3 (0.4)
Macrolides and lincosamides (J01F)	77 (10.0)
Aminoglycosides (J01G)	23 (3.0)
Quinolones (J01M)	74 (9.6)
Other antibacterials (J01X)	116 (15.0)
Antimycotics for systemic use(J02)	8 (1.0)
Antimycobacterials FOR SYSTEMIC USE (J04)	11 (1.4)
Antivirals FOR SYSTEMIC USE (J05)	8 (1.0)
Antiprotozoals (P01)	2 (0.3)

**Table 3:** Top 10 indications and antibiotics.

	Top 10 Indications		Top 10 Antibiotics	
No.	Indications	*n* (%)	Antibiotics	*n* (%)
1	Pneumonia	134 (17.3)	Ceftriaxone	116 (15.0)
2	Others	111 (14.4)	Piperacillin, enzyme inhibitor	84 (10.9)
3	SST	84 (10.9)	Metronidazole	55 (7.1)
4	Unknown	67 (8.7)	Cefuroxime	45 (5.8)
5	SEPSIS	51 (6.6)	Levofloxacin	45 (5.8)
6	OBGY	42 (5.4)	Meropenam	42 (5.4)
7	BJ	38 (4.9)	Clindamycin	41 (5.3)
8	CNS	28 (3.6)	Vancomycin	37 (4.8)
9	CVS	25 (3.2)	Cefazolin	35 (4.5)
10	P. GIT	25 (3.2)	Ciprofloxacin	25 (3.2)

BJ; Bone and Joint, CNS; Central Nervous System, GIT; Gastro-Intestinal Tract, OBGY; Obstetric or Gynaecological, P; Prophylaxis, SST; Skin and Soft Tissues.

## Data Availability

All statistical analysis was performed using IBM SPSS statistics software (IBM Corp., Armonk, NY, United States) for Windows, Version 22.0. Descriptive analyses were undertaken to calculate frequency, percentage, median, and mode. Continuous variables are expressed as the median and range.
